# Legal status as a life course determinant of health: parent status, adjudication stages, and HIV knowledge among highlanders in Thailand

**DOI:** 10.1186/s12889-021-11811-8

**Published:** 2021-10-11

**Authors:** Stephanie M. Koning, Amanda Flaim, Leo Baldiga, David A. Feingold

**Affiliations:** 1 Department of Population Health Sciences, University of Wisconsin School of Medicine and Public Health, 610 Walnut Street, 707 WARF Building, Madison, WI 53726 USA; 2 Present address: Institute for Policy Research, Northwestern University, 2040 Sheridan Road, Evanston, IL 60208 USA; 3 James Madison College and the Department of Sociology, Michigan State University, 307 S Case Hall, 842 Chestnut Rd, East Lansing, MI 48823 USA; 4 James Madison College, Michigan State University, 307 S Case Hall, 842 Chestnut Rd, East Lansing, MI 48823 USA; 5 United Nations, Educational, Scientific, and Cultural Organization (ret.)/Ophidian Research Institute, 19 Soi 35, Sukhumvit Road, Klongton-nua, Wattana, Bangkok, 10110 Thailand

**Keywords:** Legal status, Birth registration, Citizenship, Statelessness, HIV/AIDS, Health disparities, Thailand, Thai-Myanmar border, Minorities

## Abstract

**Background:**

Rising nativism and political volatility worldwide threaten to undermine hard-won achievements in human rights and public health. Risks are particularly acute for hundreds of millions of migrants, minorities, and Indigenous peoples, who face disproportionately high health burdens, including HIV/AIDS, and precarious legal status (LS). While LS is receiving increasing attention as a social determinant of health and HIV, understandings are still limited to select immigrant communities. Its effects on health among stateless communities, particularly in the Global South, remain largely unknown. Moreover, widespread limitations in census measures of LS reduce its complexity to a simplistic citizen/non-citizen binary or insufficient proxies. Thailand’s ethnolinguistically diverse highlander population experiences disproportionately high HIV prevalence and comprises one of the world’s largest and most protracted cases of statelessness, an acute condition of precarious LS. As such, analysis of LS and health outcomes among highlanders is both critically warranted, and useful as a case study outside of the migration paradigm.

**Methods:**

Drawing on the UNESCO Highland Peoples Survey II (2010), an unprecedented and unique cross-sectional census of highlanders in Thailand, we mobilize complex measures of LS in adjusted ordinal logistic regression models to assess how parent citizenship and LS adjudication over the early life course condition adult HIV knowledge—a key protective factor against transmission (*n* = 8079).

**Results:**

Adjusted ordinal logistic regression on knowledge scores reveal that parent citizenship predicts odds of greater knowledge by 1.4- to 2.2-fold, depending on ethnic group. This is partially explained by divergent stages of LS adjudication between birth and adulthood, including successful birth registration and adult citizenship acquisition, along with secondary school completion. Precisely *how* these factors contribute to HIV knowledge varies by ethnic group.

**Conclusions:**

This study advances knowledge of LS outside of the migration paradigm, reveals heretofore unexamined connections between LS and access to public health information, and elucidates how instabilities in LS adjudication stages underlie health inequalities over the life course. Findings indicate that securing success in public health and human rights agendas requires attention to how states adjudicate and deploy LS in multiple stages across the life course to structure access and exclusion among migrant and non-migrant communities alike.

**Supplementary Information:**

The online version contains supplementary material available at 10.1186/s12889-021-11811-8.

## Background

Despite considerable progress, HIV/AIDS persists as a major cause of death globally [1], disproportionately burdening marginalized groups (e.g., [2, 3]). Politically volatile conditions worldwide threaten to undermine human rights and public health achievements in HIV/AIDS eradication, particularly for minorities, Indigenous peoples, and migrants. Millions worldwide are stateless, lacking the recognition or rights of citizenship in any country, and tens of millions more experience *effective statelessness* [4], an acute condition of precarious legal status (LS)—lacking both recognition of citizenship, its attendant political and civil rights, or both [5, 6]. Recent studies have linked precarious LS to adverse impacts on healthcare access [7–11], education [12], violence against women [13], employment and migration outcomes [14–18]. However, even as LS significantly structures life and livelihoods in the twenty-first century, it remains largely overlooked in public health research, which is in part due to significant challenges in data collection and measurement.

Northern Thailand is a valuable study setting for understanding how precarious LS affects health, and HIV transmission specifically. Thailand is renowned for advancing progressive health care agendas and successfully addressing the HIV/AIDS epidemic [19–21]. Yet, the benefits of the HIV/AIDS prevention campaign have not been fully realized by highlanders; a diverse population of ethnic minorities and Indigenous communities who predominantly live in the mountainous North. Highlanders disproportionately experience both high HIV prevalence and low awareness of HIV transmission relative to lowland ethnic Thais [22, 23]. This is due, in part, to persistent educational barriers for highlander youth and failures to provide culturally and linguistically appropriate information [24, 25]. Highlanders have also been subjected to precarious LS and protracted statelessness over decades. Yet, how statelessness and precarious LS underlie the HIV/AIDS burden remains underexamined beyond studies of relatively narrow outcomes, such as drug use initiation among select groups [25]. Studying a general HIV risk factor, like accurate knowledge of transmission, in a more representative sample substantially expands current understandings of the problem and identifies structural solutions.

The current study reveals that associations between LS and HIV knowledge are more complex than currently theorized. Specifically, we uncover inconsistencies and instabilities in stages of LS adjudication over the life course—from parent citizenship to birth registration to adult citizenship confirmation—and education that significantly influence HIV knowledge in adulthood. Moreover, we detect significant ethnic differences in these relationships. These findings inform understandings of LS as a multidimensional and dynamic health determinant. Meanwhile, states around the world variously enact and reinforce precarious LS across and between generations of marginalized communities—e.g., through burdensome and exclusionary evidentiary requirements [4, 26].

Our theoretical and empirical contributions join multiple recent calls for attention to LS in public health and medicine [5, 11, 27, 28]. Our conceptual model is the first to specify LS adjudication stages and health implications that correspond widely to other states’ evidentiary requirements, including in contexts where the LS of marginalized individuals or groups can remain precarious as they remain in their country of birth over the life course. Thus, it is adaptable for studying precarious LS and health outcomes in other settings. Below, we elaborate on how LS is a social determinant of health and HIV, why highlanders in Thailand present an illuminating study setting, and the current study’s approach.

### Legal status as a health determinant

Tens of millions of people worldwide are subjected to precarious LS by states that neglect to, or refuse to, recognize them as citizens or otherwise full, rights-bearing residents. Millions are *de jure* stateless [6, 29]— lacking a legal claim to citizenship in any country. Millions more are *effectively* stateless, meaning they lack *recognition* of citizenship, and access to its attendant rights [4]. Precarious LS extends across generations when the country of birth alone does not automatically confer the recognition and rights of citizenship (*jus soli*), but jointly requires citizenship of the parent (*jus sanguinis*)—as in Thailand. Whereas precarious status and statelessness are widespread in parts of the Global South, data gaps and research risks preclude studies of status and related outcomes. As a result, the vast majority of research on LS and health focuses on (im) migrant communities in the Global North. Nevertheless, these studies enable a critical framing of how precarious LS and statelessness can directly or indirectly affect health and health knowledge in communities like highlanders in Thailand.

Beyond the health impacts of LS covered in prior studies, primarily on legal barriers to insurance coverage and government services [4, 5, 8, 30, 31], non-citizens without documentation must weigh healthcare needs against fears of encountering stigma in health care settings [27, 32–34], arrest or deportation [35], or both [8, 36, 37]. These fears are substantiated by documented racism and bureaucratically mandated reporting of LS in clinical settings [30, 38, 39]. Indirect barriers stem from unstable employment, lower incomes, and language barriers [32, 38, 40, 41].

LS may further act as a structural determinant of health, via poverty, educational attainment, or social position [42]. Specifically, precarious LS contributes to social and environmental factors consequential for health: i.e.*,* restricted mobility, human rights abuses, and poorer educational opportunities [13, 43]. Thus, LS may shape individual health trajectories and population health inequalities that extend intergenerationally. Moreover, social perceptions of who has, or “deserves” LS, may produce spillover health effects among vulnerable and stigmatized racialized groups more broadly [27, 44, 45]. The health impacts of similar forms of structural and interpersonal discrimination are well-documented, and often mediated through inequalities in health-related environments, resources, and safety nets [46–48].

### Legal status and HIV

The ongoing HIV/AIDS pandemic remains a devastating global health crisis that both structures, and is structured by, persistent social inequities worldwide (e.g., [49–53]). Yet very few studies have examined the specific role of LS in structuring HIV/AIDS risk. Prior work highlights direct ways that precarious LS is associated with inconsistent access to HIV testing, counseling, and therapeutic services, and findings on HIV status remain mixed [54–57]. Still, very few studies have been able to assess precarious LS as an underlying, structural determinant of HIV infection risk; nor has the relationship between LS and HIV risk been investigated in large, population-based data from areas substantially burdened by precarious LS like statelessness.

Beyond stateless and Indigenous populations being underrepresented in global health research generally, HIV status is often unknown and testing prohibitively expensive in these contexts. Thus, we focus the current study on assessing accurate knowledge of HIV transmission as a necessary step for measuring capacity for protective behavior and infection risk. Although an incomplete measure of risk alone, accurate HIV transmission knowledge has been shown to predict more protective behavior and perceptions of risk [58–61].

Our study builds on this prior work to contribute a greater understanding of how precarious LS structures HIV risk, by revealing how it structures HIV knowledge in a population acutely affected. In addition to assessing this association overall in the highlander population, we offer a fuller model of how LS early in life fundamentally contributes to intermediate social factors that are already known to be influential for accurate HIV knowledge. We focus specifically on educational attainment in a rural setting affected by high unemployment, adjusting for age and cohort. Educational attainment is not only an established predictor of HIV knowledge that predicts likelihood of testing and serostatus awareness [58, 60, 62, 63]; it is also significantly associated with parental LS in the highland context [15, 64]. Before elaborating further on the study design, we review relevant historical background for the current situation of statelessness and HIV/AIDS among highlanders below.

### Highlanders, statelessness, and HIV/AIDS in northern Thailand

Highlanders in Thailand have faced the dual burdens of HIV/AIDS prevalence and precarious LS for decades. Understanding the interconnected nature of this dual burden requires attention to historical population changes in a complex border zone. However, the deep roots of these burdens also lie in the Thai state’s long-standing exclusion of highlanders and its disruption of their traditional livelihoods.

Highlanders in Thailand comprise a diverse and dynamic population of distinct ethnic communities. However, they are poorly represented and vastly underestimated in population data, because ethnicity is omitted from the Thai census. Karen, Akha, Lahu, Lisu, Khmu, Mien, H’tin, Hmong, and Lua people – groups referred to pejoratively in both official state policy and Thai public discourse as “hill tribes”—were last estimated in 1996 at nearly two million people [65]; yet the highlander population is larger and more diverse than these communities alone. The United Nations Educational, Scientific, and Cultural Organization Highland Peoples Survey II (UNESCO HPSII), the most recent census of highland villages (described in detail below), reflects a mosaic of more than 18 ethnic groups and an extremely complex picture of LS, with more than 25% of highlanders experiencing precarious LS linked to statelessness or limited residency rights [15, 43].

Thailand primarily recognizes rights to citizenship by parentage (jus sanguinis), but also recognizes rights to citizenship by birthright (jus soli) for those who can demonstrate proof of one’s birth in the country—or the birth of one biological parent in the country [15, 43]. Citizenship, in Thailand, is thus adjudicated in three interconnected stages: A child of a Thai citizen (1) will be registered at birth (2); and, at the age of 15, the child is issued a citizen ID that confirms the child’s status as citizen (3). Documentation of parentage, birthplace, and other related evidence are used to substantiate these claims at each stage of LS adjudication. For highlanders, however, the problem lies in the evidence, or a lack thereof. Whether by purposeful exclusion, neglect, or a combination of both, uneven state projects of the “hill tribe” registration and ‘development’ have produced vast gaps and inconsistencies in evidence across generations that continue to be read by officials as lack of eligibility for Thai citizenship [43].

Evidentiary gaps in the highlands are rooted in a politics of omission: Highlanders had long been excluded from Thai civil registries and citizenship until the 1960s [66–69]. Amidst regional upheaval in the wake of anti-colonial struggle, the Thai state turned its attention toward its borderlands and resident highlanders, whose ‘non-Thai’ identities and mobile agricultural practices were increasingly deemed threats to national security. The state’s narrative of “‘hill tribe’ problems,” which included threats of communist insurrection, drug/opium trafficking, and forest destruction [70–74], was mobilized to justify the perpetual exclusion of highlanders from Thai citizenship and increasingly draconian control over their lives and livelihoods.

Various surveys of the “hill tribe” population, implemented between the 1960s through the late 1990s, anchored Thailand’s politics of exclusion and control [75]. Registrations served state interests of documenting and controlling the population rather than recognizing highlanders’ rights to citizenship. As few highlanders could read Thai or understood the importance of saving survey documents during this period, compiling or accessing sufficient evidence of residency or birth was a difficult-to-impossible task [76]. Indeed, until the turn of the millennium, the vast majority of highlanders were born at home in their villages [15]; and very few understood the importance of birth registration for their children’s futures [76]. Moreover, reports of discrimination by district officials discouraged highlanders from attempting to register their children at birth [15, 76]. Indeed, common practices of harassment, extortion, arbitrary arrest, and even deportation of highlanders at internal border checkpoints deterred many highlanders from leaving home to register their children at birth or registering themselves as residents of the state [15, 76, 77]. Finally, extremely few highlanders were literate in Thai [15], as schools in the highlands were operated inconsistently from the 1960s through the 1980s and often seen by local communities as irrelevant to highland life [15, 64, 76]. By the late 1990s, when the importance of citizenship for highlanders’ livelihoods, safety, and futures became critically clear, children who lacked birth registration were nevertheless denied access to free education [64]. And, although highlanders mobilized for recognition of their citizenship at the turn of the millennium, as many as 20% of highlanders who are citizens by law are nevertheless subjected to protracted statelessness, the rates of which vary substantially by highlander subgroup [43].

The factors that have produced the burden of precarious LS among highlanders are similarly linked to disproportionately high HIV/AIDS prevalence. Just as the narrative of “‘hill tribe’ problems” justified the continued exclusion of highlanders from citizenship, this same narrative justified the widespread disruption of highlanders’ livelihoods in the name of opium eradication. With U.S. and international pressure and funding, the Thai government initiated a vast program of opium suppression in the highlands—an ill-conceived and poorly executed program that came at significant costs to the health and well-being of highlanders [75]. Devastated livelihoods, displacement, and the spread of heroin addiction posed both direct and indirect risks related to the HIV/AIDS crisis in the highlands. The delayed national response to HIV/AIDS in the highlands exacerbated the crisis as well [78, 79].

One of the only population-based studies in the 1990’s estimated HIV prevalence among ethnic minorities in northern Thailand to be 2.13%, including high prevalence among Akha (5.0%) and Lahu (0.63%) villages, and low prevalence among Karen villages (0.0%) [22]. Risk has been closely related to some highlander subgroups’ disproportionate representation in jobs associated with injection drug use and sex work in the 1990s and 2000s [22, 23, 32, 80, 81]. Although public health outreach has improved, highlander subgroups continue to face distinct and overlapping forms of discrimination, stigma, and linguistic and social barriers preventing them from reaching HIV-related services and accurate information [40, 82–86]. Thus, more careful attention to specific ethnocultural context is necessary for better understanding how to overcome subgroup-specific barriers.

While prior work strongly suggests these broad structural determinants linking LS to HIV/AIDS in the highlands, many important mechanisms underlying these relationships over the life course remain understudied, including education. Precarious LS early in life has historically hindered educational attainment among highlanders. This has occurred through direct and indirect ways, including LS documents being required for individual enrollment and/or schools in communities with high levels of statelessness being under-resourced or under-staffed [64, 87–89]. More indirect pathways involve state restrictions on land, mobility, and livelihoods that contribute heavily to wealth disparities along LS lines [77]. This can pose barriers to educational enrollment and attainment among children in affected households due to competing demands to work, provide childcare, or cover other indirect costs related to school attendance. In turn, education has been critical for acquiring citizenship among highlanders, particularly by facilitating Thai language learning and connecting students to potential advocates in schools that, in some cases, can help offer support in citizenship claims [15]. Prior ethnographic work suggests that these barriers have also been uneven across highlander subgroups over recent history, warranting careful comparisons. In sum, many direct and indirect relationships reinforce the connection between LS and education. This relationship likely results in sizeable impacts on HIV knowledge and related inequalities, particularly considering how education is one of the strongest and most consistent predictors of HIV knowledge [90–93].

### Study objectives and approach

As demonstrated above, LS in Thailand, as in many other contexts, is not a simple citizen/non-citizen binary. It is, rather, adjudicated at multiple life stages from parental status, to birth registration, to citizenship confirmation. Yet, in a context of vast evidentiary gaps and discrimination, the path between these stages is never given. Our study therefore offers a framework of LS as a structural determinant of health that is not necessarily stable across the life course, nor intergenerationally.

**Fig. 1:**
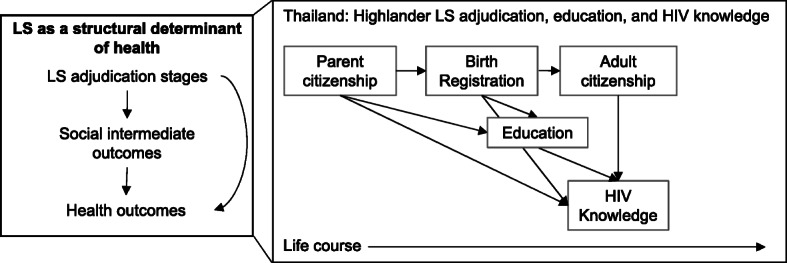
Legal Status (LS) adjudication as a structural, life-course determinant of health: A conceptual framework of how LS adjudication affects health (left), and a scaled-down model of how this contributes to inequalities in HIV protection among highlanders in Thailand (right)

Figure 1 presents a conceptual framework (left panel) alongside a scaled-in examination of how stages of LS adjudication are experienced among highlanders in Thailand (right panel). Both panels depict LS stages in the top row, social intermediate outcomes in the middle, and health outcomes at the bottom. The right panel depicts time over the life course from left to right with other measures specific to the current study analysis, including HIV knowledge as the outcome important for ultimate HIV protection. Because Thai citizenship is primarily determined by evidentiary requirements at different life stages, our Thailand model includes life-stage-specific LS and documentation: parent citizenship, birth registration, and adult citizenship (confirmed at age 15). This representation highlights how individual LS is dynamic over the life course: due to strict legal evidentiary requirements—citizenship can be recognized or denied when evidentiary proof or gaps, respectively, are encountered at one or more adjudication stage. For example, before age 15, parent citizenship (identification cards) and birth registration (birth certificates) are the primary documents that confer secure LS. After age 15, one’s own government-issued identification card becomes the primary LS-conferring document. We include educational attainment as an intermediate factor linking LS to knowledge across life stages, along with direct arrows encompassing other pathways.

We assess evidence supporting this model using unprecedented data from the highlands. First, we model how adult HIV knowledge is associated with parent citizenship—the earliest stage of LS adjudication measured in our study. In this step, we assess differences by highlander ethnicity. Due to the historically strong association between LS adjudication, ethnic-based discrimination, and other social barriers, we expect that differences in cultural and historical context within the broader ‘highlander’ group moderate multiple associations of interest. This includes differences in the localized barriers and social stigmas that distinct highlander groups have historically encountered related to civil registration, education, and health.

Second, we stage a series of regression analyses to assess the independent and related roles of each individual LS adjudication stage on HIV knowledge, and how differences in HIV knowledge by parent citizenship are explained by intermediate LS adjudication—birth registration and adult citizenship—and education. This approach allows us to assess whether the impact of parent citizenship on adult HIV knowledge persists or is diminished based on subsequent LS acquisition and whether specific stages of the relatively dynamic LS adjudication process, and related social outcomes, mediate this relationship. Mediation would be evidenced by the attenuation of this association after adjusting for these intermediate variables.

## Methods

### Survey data

The study data were derived from the UNESCO HPS II, conducted in 2010 in border villages located in five northernmost and northwesternmost provinces. Funded by UNESCO and implemented by the Royal Thai Government’s Bureau of Social Development (BSD), it is the most comprehensive and expansive survey on statelessness conducted in the world. It was designed to measure complex dynamics of LS and understand the effects of statelessness on a range of life and livelihood outcomes. It substantially expanded the coverage of villages relative to the HPS I (2005), and includes information on multiple, unique measures of LS, as well as education, migration, family relationships, and health in the highlands. Sampling criteria were based on village proximity to the international border (< 20 km). All households within villages in Chiang Rai, Chiang Mai, and Mae Hong Son provinces were sampled (*n* = 179), along with 25% of households in villages in Tak and Kanchanaburi provinces (*n* = 113). The sampling frame, generated by the Center for Hill Tribe Development and Welfare, was a full roster of highland villages. The HPS II sample included 292 villages and 15,396 households. The overall response rate was exceptionally high (99%), which was attributed to villagers’ keen interest in LS issues [15].

Questionnaires were administered in person by trained BSD staff to adult representatives of each household and are published in the original data collection study [14]. The data collection protocol followed contemporary UNESCO ethical guidelines for human subjects research among indigenous peoples, with informed consent in local languages provided in two venues. First, prior to implementing each survey, surveyors met with villagers in open fora to explain, in detail, the objectives and limitations of the survey, enabling community members and representatives to ask clarifying questions and to register concerns about the survey in a collective, safe environment. Second, oral consent was acquired by each individual participant prior to initiating surveys. Women were encouraged to be representatives/respondents. Interviews were conducted in a space of each respondent’s choosing and in respondents’ preferred languages, including highlander languages and northern Thai, each of which were pretested. Upon data entry all data were completely de-identified. The current analysis of these anonymized data was completed at University of Wisconsin (UW) as non-human subjects research, with exemption status confirmed by the UW Institutional Review Board.

The current study analysis is limited to Lahu, Karen, Akha, Hmong, Lisu, and Mien people—based on self-reported ethnicity—due to subsample size. We limited the analysis to individuals who reported being born in Thailand to focus on structural barriers specific to LS adjudication in Thailand over the life course. The current study analysis used de-identified data at University of Wisconsin (UW) as non-human subjects research, with exemption status confirmed by the UW Institutional Review Board.

### Measures

Respondents’ HIV knowledge scores were calculated as sums of correct responses to questions regarding HIV transmission, adapted from the cross-culturally validated Demographic and Health Survey instrument (2008–2013). This included the following separate questions: “Can you contract HIV/AIDS from: sharing a needle with someone who has HIV/AIDS; a mosquito or insect bite; from a mother’s womb to her child; eating with someone who has HIV/AIDS; having sex with someone who has HIV/AIDS without using a condom; kissing a person who has HIV/AIDS?”

The survey requested information on each parent’s citizenship status, including those deceased and separated at the time of the survey. We constructed a single variable to indicate whether at least one parent had citizenship. The following binary variables measured intermediate stages of LS adjudication: birth registration and adult citizenship documentation. Education is also included as an intermediate variable and categorized by years of school completion: no school, some primary (1–6 years), primary complete (6–8 years), lower secondary (9–11 years, which fulfills the minimum level of compulsory schooling in Thailand), and upper secondary (> 12 years).

We included wealth quartile categories, based on a factor analysis of house materials [77], and age as a continuous variable in adjusted models.

Table 1 summarizes sample characteristics, comparing highlanders and ethnic Thais. Missingness for all analytical variables ranged from 0 to 5% (online supplement; Table A1).

**Table 1:** Comparison of highlander and ethnic Thai HPS II respondents. Results for tests of different means (t-tests) and proportions (X^2^ tests) indicated as: ***p < 0.001, **p < 0.01, *p < 0.05

	Highlander	Thai	
Total (n)	8079	1866	
HIV Knowledge Score (0–6)			
Mean	3.1	4.2	***
Median	3	5	
100% correct	19.5%	30.4%	***
LS adjudication			
Parent with citizenship	71.3%	96.1%	***
Birth registered	31.2%	73.4%	***
Adult citizenship	86.5%	98.8%	
Highest education level completed			
Less than primary	80.1%	64.4%	***
Primary	11.0%	18.8%	***
Secondary	8.9%	16.8%	***
Age			
17–25	12.1%	5.4%	***
26–35	24.8%	14.7%	***
36–45	27.0%	23.5%	***
46–55	19.2%	26.3%	***
56+	17.0%	30.2%	***
Male sex	58.7%	55.8%	*
Ethnicity			
Karen	43.9%	–	
Lahu	30.5%	–	
Akha	13.0%	–	
Hmong	9.5%	–	
Lisu	3.2%	–	
Wealth quartile			
1	33.5%	3.7%	***
2	24.0%	29.9%	***
3	25.4%	36.7%	***
4	17.1%	29.7%	***

### Analysis

We first compared HIV knowledge, parent citizenship, and other characteristics between highlander and ethnic Thai respondents (Table 1). We also detail differences in specific question responses by ethnic categorization and parent citizenship jointly online (Figure. A1). To assess stability of LS across adjudication stages, staring with parent citizenship, we assessed differences by parent citizenship in birth registration, school completion, and adult citizenship (Table 2). In our final models, we excluded ethnic Thai respondents because nearly all had a parent with citizenship.

**Table 2:** Legal status adjudication and secondary school completion by parent citizenship among highlander HPS II respondents. X^2^ test results indicated as: ***p < 0.001, **p < 0.01, *p < 0.05

	Citizen Parent	No citizen parent	
Birth registered	41.0%	6.6%	***
Secondary school completed	11.1%	3.9%	***
Adult citizenship card	98.0%	57.6%	***

To model differences by parent citizenship, we performed ordinal logistic regression. This is a generalized linear mixed model that is optimized for ordinal scores, like our HIV knowledge measure, and allows for nonlinearity. We deployed it using likelihood estimation with Laplace approximation, in SAS 9 with PROC GLIMMIX. We elaborate on our model selection and specification in the online methods supplement, including how we account for village-level variation using village random intercepts. We adjusted for respondent sex and age. To account for differences in the impact of parent citizenship on HIV knowledge by ethnicity, we included interaction terms between parent citizenship and indicators for each ethnic group, which were statistically significant (Table A2 online; Likelihood ratio: Χ^2^(4) = 17.0, *p* = 0.002).

To further investigate whether and how respondents’ prior birth registration, educational attainment, and adult citizenship explained differences by parent citizenship we built staged ordinal regressions to estimate: (1) the total association between HIV knowledge and parent citizenship, and (2) attenuation of this association attributable to subsequent LS and education to indicate potential mediation in the following order: birth registration, school completion, and adult citizenship documentation. Based on the evidence that LS is differentially associated with HIV by ethnicity, we performed this analysis separately for the three largest ethnic groups in the sample (Karen, Lahu, and Akha). To isolate differences attributable to experiences of LS adjudication beyond what may be driven by cohort and age differences, we limited each subgroup analysis to respondents 35 years old and younger.

To further visualize attenuation patterns of the parent citizenship association with HIV knowledge, we also plotted subgroup comparisons across estimated HIV knowledge scores. Here, we use estimates from a second set of staged regressions that allowed for two-way interaction terms between parent citizenship and each intermediate variable to allow for more in depth subgroup comparisons (online Figure A2). Here, we estimated conditional predicted probabilities for obtaining each HIV knowledge score and then plotted probability ratios between respondents with and without a citizen parent, averaged across men and women and conditioned on mean age and median wealth. We then recalculated these estimates after adding and conditioning on each subsequent intermediate variable.

## Results

### Parent legal status and HIV knowledge

First, highlander and ethnic Thai respondents living in similarly remote border villages possess very different levels of HIV knowledge, with mean HIV scores of 3.1 and 4.2 and median values of 3 and 5 among highlander and Thai respondents, respectively (Table 1). Across individual knowledge questions, ethnic Thai respondents are consistently more likely to answer questions correctly than highlander respondents; and, among highlanders, those with parent citizenship are more likely to answer questions correctly (Figure A1). There are also stark differences between highland and Thai respondents in LS adjudication indicators with 71.3 and 96.1% with parent citizenship, 31.2 and 63.4% with birth registration, and 86.5 and 98.8% adult citizenship documents, respectively. Thai respondents were also nearly twice as likely to complete upper secondary school.

Second, Table 2 reveals multiple instabilities in the linkages between parent citizenship among highlanders across life-course LS adjudication stages and education. Among individuals with a citizen parent, only less than half have birth registration and 11.1% completed secondary school. However, 98% have adult citizenship. In contrast, individuals without a citizen parent are 84% less likely to have birth registration, 65% less likely to complete lower secondary school, and 41% less likely to have adult citizenship documentation.

Results from our initial model assessing the adjusted association between parent citizenship and HIV knowledge among highlanders revealed that respondents with at least one citizen parent indeed achieved higher HIV knowledge scores (online Table A2). Model point estimates are left on the logit scale due to model interactions rendering main term coefficients uninterpretable when transformed to odds ratios, but the direction of the logit coefficients remain interpretable. We see that older age is associated with lower HIV knowledge. Being male and wealthier is associated with higher HIV knowledge. Variation in the HIV knowledge advantage associated with parent citizenship by ethnic group is reflected in respective interaction coefficients. For instance, relative to Karen respondents, other groups experienced a smaller advantage associated with parent citizenship. This was in addition to the predominantly negative main effects associated with belonging to a non-Karen ethnic minority group.

### Parent citizenship and intermediate social determinants: birth registration, education, and adult citizenship

Based on the significant interactions between parent citizenship and ethnicity uncovered, we stratified our final staged regressions by ethnicity. Table 3 presents the results of each regression in separate columns and results from separate ethnic groups (Karen, Lahu, and Akha) in separate rows. HIV score odds represent the estimated odds of each score threshold. Among Karen respondents, those without a parent with citizenship were estimated as having an adjusted odds of answering at least 1 question correctly of 2.21 and an adjusted odds of answering 6 questions correctly of 0.13. Estimated odds ratios and 95% confidence intervals are also included. Among Karen respondents, having at least one citizen parent was associated with 2.19 greater odds of achieving a greater HIV score relative to a respondent without a citizen parent across score thresholds. Parent citizenship was similarly associated with higher HIV knowledge among Lahu and Akha respondents.

**Table 3:** Odds and odds ratio point estimates (PE) from staged ordinal logistic regressions with 95% confidence intervals (CI), stratified by ethnic subgroup for three largest ethnic groups represented in HPS II

	PE	95% CI		PE	95% CI		PE	95% CI		PE	95% CI	
*Karen (n = 1288)*												
**HIV SCORE ODDS**												
> 1	2.21	(1.35,	3.60)	2.13	(1.31,	3.47)	1.49	(0.93,	2.41)	1.58	(0.97,	2.59)
> 2	1.89	(1.16,	3.08)	1.81	(1.11,	2.95)	1.26	(0.78,	2.03)	1.33	(0.81,	2.18)
> 3	1.43	(0.88,	2.34)	1.38	(0.85,	2.24)	0.94	(0.58,	1.51)	0.99	(0.61,	1.62)
> 4	0.78	(0.48,	1.27)	0.74	(0.46,	1.21)	0.50	(0.31,	0.80)	0.53	(0.32,	0.86)
> 5	0.50	(0.31,	0.81)	0.47	(0.29,	0.77)	0.31	(0.19,	0.51)	0.34	(0.20,	0.55)
> 6	0.13	(0.08,	0.22)	0.13	(0.08,	0.21)	0.08	(0.05,	0.14)	0.09	(0.05,	0.15)
**ODDS RATIOS**												
**Parent citizenship**												
At least one parent with citizenship	2.19	(1.59,	3.02)	1.69	(1.19,	2.41)	1.49	(1.04,	2.12)	1.83	(1.07,	3.12)
**Birth registration**												
Yes				1.50	(1.15,	1.96)	1.27	(0.97,	1.67)	1.32	(1.00,	1.74)
**Education**												
No school							(Reference)			(Reference)		
Some primary school							1.57	(1.15,	2.15)	1.60	(1.16,	2.19)
Primary complete							3.14	(2.30,	4.28)	3.15	(2.31,	4.30)
Lower secondary complete							3.62	(2.41,	5.43)	3.63	(2.42,	5.45)
Upper secondary/ vocational complete							3.85	(2.43,	6.08)	3.87	(2.45,	6.13)
**Adult citizenship**												
Yes										0.72	(0.40,	1.32)
*Lahu (n = 825)*												
**HIV SCORE ODDS**												
≥ 1	3.14	(1.69,	5.83)	3.02	(1.66,	5.50)	2.54	(1.38,	4.66)	2.19	(1.09,	4.38)
≥ 2	2.04	(1.10,	3.77)	1.99	(1.09,	3.61)	1.65	(0.90,	3.02)	1.41	(0.71,	2.83)
≥ 3	1.17	(0.63,	2.17)	1.15	(0.63,	2.08)	0.93	(0.51,	1.70)	0.80	(0.40,	1.61)
≥ 4	0.56	(0.03,	1.04)	0.54	(0.30,	0.98)	0.43	(0.23,	0.78)	0.37	(0.18,	0.74)
≥ 5	0.29	(0.16,	0.54)	0.28	(0.15,	0.50)	0.21	(0.11,	0.39)	0.19	(0.09,	0.38)
≥ 6	0.10	(0.05,	0.18)	0.10	(0.05,	0.18)	0.07	(0.04,	0.14)	0.06	(0.03,	0.13)
**ODDS RATIOS**												
**Parent citizenship**												
At least one parent with citizenship	1.52	(1.11,	2.09)	1.61	(1.17,	2.21)	1.41	(1.02,	1.96)	1.15	(0.77,	1.71)
**Birth registration**												
Yes				1.00	(0.71,	1.41)	0.84	(0.59,	1.20)	0.79	(0.55,	1.14)
**Education**												
No school							(Reference)			(Reference)		
Some primary school							1.71	(1.14,	2.56)	1.75	(1.16,	2.62)
Primary complete							2.51	(1.68,	3.73)	2.25	(1.50,	3.36)
Lower secondary complete							2.71	(1.70,	4.32)	2.79	(1.73,	4.50)
**Adult citizenship**												
Yes										1.53	(0.97,	2.44)
*Akha (n = 315)*												
**HIV SCORE ODDS**												
≥ 1	5.56	(2.52,	12.28)	4.31	(2.03,	9.14)	3.03	(1.57,	5.82)	3.17	(1.67,	6.02)
≥ 2	4.49	(2.05,	9.83)	3.51	(1.82,	6.79)	2.42	(1.27,	4.61)	2.54	(1.35,	4.77)
≥ 3	2.2	(1.02,	4.75)	1.67	(1.08,	2.59)	1.13	(0.60,	2.10)	1.18	(0.64,	2.17)
≥ 4	0.95	(0.45,	2.02)	0.73	(0.58,	0.92)	0.48	(0.26,	0.89)	0.50	(0.27,	0.91)
≥ 5	0.44	(0.21,	0.93)	0.33	(0.33,	0.33)	0.21	(0.11,	0.40)	0.22	(0.12,	0.41)
≥ 6	0.22	(0.10,	0.47)	0.17	(0.10,	0.29)	0.11	(0.06,	0.21)	0.11	(0.06,	0.21)
**ODDS RATIOS**												
**Parent citizenship**												
At least one parent with citizenship	1.43	(0.94,	2.17)	1.44	(0.96,	2.16)	1.17	(0.74,	1.84)	1.20	(0.73,	1.97)
**Birth registration**												
Yes				1.10	(0.53,	2.27)	1.02	(0.54,	1.91)	1.04	(0.55,	1.97)
**Education**												
No school							(Reference)			(Reference)		
Some primary school							1.83	(0.87,	3.85)	1.82	(0.85,	3.86)
Primary complete							3.00	(1.63,	5.54)	2.89	(1.55,	5.37)
Lower secondary complete							2.69	(1.43,	5.03)	2.67	(1.42,	5.01)
Upper secondary/ vocational complete							3.91	(1.78,	8.59)	3.76	(1.67,	8.44)
**Adult citizenship**												
Yes										0.94	(0.51,	1.73)

As expected, birth registration, educational attainment, and adult citizenship were each independently associated with HIV knowledge and partially accounted for the association between parent citizenship and HIV knowledge. However, the strength of these associations and how they relate varied by ethnic group. Among Karen respondents, birth registration was associated with 50% greater odds of higher HIV knowledge and accounted for 42% of the difference associated with parent citizenship. Completed schooling was further associated with greater HIV knowledge and accounted for an additional 29% of the parent citizenship advantage. Birth registration was no longer associated with HIV knowledge after accounting for education. Adult citizenship was not associated with HIV knowledge, but other LS adjudication stage predictors strengthened when it was added.

Among Lahu respondents, there was a gradual attenuation of the parent citizenship association with HIV knowledge (OR = 1.52; 95% CI = 1.11, 2.09) as each intermediate variable was added. The exception was birth registration, which had no apparent association with HIV knowledge. Education strongly predicted HIV knowledge, and accounted for 33% of the parent citizenship advantage, beyond birth registration. Including adult citizenship accounts for an additional 63% of the association, beyond education. However, the individual coefficients for LS adjudication stages are not significant in this group.

Among Akha respondents, parent citizenship was possibly associated with greater HIV knowledge, but with a wider confidence interval (OR = 1.43; 95% CI = 0.94, 2.17). This was attenuated almost entirely by education, by 95%.

The additional subgroup comparisons by individual HIV knowledge scores and across staged model estimations (online Figure A2) agree with Table 3, showing additional detail. Figure A2 plots probability ratios between respondents with and without a citizen parent, calculated before and after adding and conditioning on each subsequent intermediate variable. It shows that among Karen people, parent citizenship was associated with an 88% greater probability of a perfect HIV knowledge score. In comparison, parent citizenship was associated with a 45 and 32% greater probability of a perfect score among Lahu and Akha respondents, respectively. Eighty-one percent of the parent citizenship advantage in answering all HIV questions correctly among Karen respondents was explained by birth registration, educational attainment, and adult citizenship. Among Lahu and Akha respondents, the same intermediate variables accounted for 47 and 97%.

## Discussion

HIV transmission knowledge is a critical resource for minimizing infection risks. For communities experiencing high HIV prevalence, like highlanders in Thailand, this resource remains dangerously elusive [40, 85]. Statelessness and precarious LS have largely been ignored as contributors to this problem, among highlanders and generally. Our study demonstrates the fundamental role of LS as a complex social condition that shapes health-relevant exposures over the life course, starting with parent LS. Specifically, this research challenges conventional methodological and theoretical models that frame LS as a static binary of citizen/non-citizen. Furthermore, it expands understandings of social and cultural disparities in HIV knowledge gaps, and potentially in health inequalities more broadly.

Importantly, our study found LS to be neither simple nor simply associated with accurate HIV/AIDS knowledge. Rather, parent LS structured HIV knowledge via subsequently linked, yet unstable, stages of the adjudication process: birth registration certification and adult citizenship. By conceptualizing LS as a series of stages, we revealed instabilities therein, which corresponded to differential influence on HIV knowledge and education. First, we found an enduring association between parent citizenship and adult HIV knowledge, independent of age, sex, and wealth. This association was attenuated by birth registration and by official confirmation of citizenship. This suggests that parent citizenship has an enduring impact on adult health through multiple stage of LS determination and educational opportunities over the early life-course, which each pose independent and cumulative impacts on HIV knowledge. However, these signs of potential mediation were partial and there were still lasting impacts of parent citizenship, or lack thereof, depending on ethnic subgroup. This finding suggests that LS carries influence both over the individual life course *and* across generations. These implicated pathways will likely be pivotal in the perpetuation or amelioration of future HIV inequalities. For instance, even amid increasing access to adult citizenship among some groups, there are still enduring associations between HIV knowledge and earlier life LS adjudication and education access.

We further examine how parent LS and subsequent adjudication stages variously structured HIV knowledge via barriers to education and other resources early in life. Prior research has demonstrated the protective role of educational attainment in promoting personal HIV knowledge and HIV risk reduction [41, 94]. Additionally, parent citizenship and birth registration have been shown to be highly predictive of educational attainment among highlanders in Thailand [15, 64]. To our knowledge, this study is the first to connect these pathways. Evidence from all three of the largest highlander groups indicated that educational attainment accounted for 29–95% of the HIV knowledge advantage attributed to parent citizenship. Other mediators that link parent citizenship and knowledge remain largely unstudied and may include education quality, access to health information through educators or otherwise informed social networks, and mobility.

Finally, our study revealed significant ethnic differences in HIV knowledge and its association with LS adjudication. Lahu, Akha, Hmong, and Lisu respondents were less likely than Karen respondents to answer HIV transmission questions correctly. Prior studies have similarly documented differences in HIV knowledge between ethnic minorities in northern Thailand [40, 85], yet none assess these differences by citizenship. First, the parent citizen advantage was weaker among Lahu and Akha respondents, compared to Karen respondents. Second, this advantage was explained by subsequent LS adjudication stages to different degrees between ethnic subgroups. These different patterns of attenuation could be explained by ethnic differences in how strongly parent citizenship predicts subsequent LS adjudication, educational attainment, or both. They could also be explained by LS or educational attainment being more predictive of adult HIV knowledge among certain ethnic groups. More research is needed to contextualize these findings and better understand how LS may inform, exacerbate, or buffer other barriers related to ethnicity, culture, and language. This need is further supported by prior research on statelessness in Thailand with respect to variations in ethnic groups’ experiences with civil registration and identification policies, land dispossession, access to education, free mobility, and safe, equitable employment compensation [43, 77, 88, 95, 96].

### Limitations

This work is not without limitations. First, HIV knowledge as the study outcome was not a direct measure of risk and is incomplete on its own. Future work should expand on other HIV-related outcomes, and health more generally. Second, because findings from this study derived from cross-sectional survey data, associations were correlational rather than causal. It is possible that the association between LS and education was bidirectional. In some cases, educational attainment may have affected parent citizenship. Ethnographic evidence suggests this is plausible but not likely driving estimates [15, 76]. Authorities are discouraged from recognizing the citizenship of anyone who cannot prove their birth in the country, thus undermining claims of older generations who were mostly born at home and lack certification of birth or early residence [77]. Still, the limitations of the current study warrant future research, including mediation pathway analyses with updated and longitudinal data, that would further elucidate mechanisms linking LS and HIV/health over the life course among affected populations, families, and individuals. This continued work is critical for understanding the complexity of specific situations and adequately informing future interventions and policy.

Additional study limitations relate to respondent selection. Due to resource limitations and the survey design, only one household representative answered the HIV questions. Thus, the generalizability of findings is limited. However, the respondents still comprise an important subpopulation: household leaders and parents of the next generation. Based on the survey sampling, findings may not be generalizable to other minority, Indigenous, and stateless people in more urban settings either: e.g.*,* where LS may not similarly constrain access to health information. Regardless of these limits to generalizability, understanding how LS affected health and knowledge in this border population remains critically important. Indeed, the authors’ 2019 ethnographic research with highlanders and interviews with highlander NGOs indicate that, while our data represents knowledge dating back to 2010, little is changing with respect to LS and related HIV/AIDS concerns in the highlands.

## Conclusions

Growing global concerns regarding statelessness and precarious LS carry grave implications in the ongoing HIV/AIDS epidemic, and health and human rights more broadly. Yet, this issue is often obscured when LS is theorized as a simple measure and siloed into immigrant health research. Our study highlights the health implications of instabilities in LS adjudication relevant to numerous contexts beyond the migration paradigm. In doing so, we offer a foundational analysis and adaptable approach for future studies that focuses in greater detail on context-specific mechanisms over the life course. This is especially relevant with the emergence and re-emergence of authoritarian regimes and related political volatility, alongside enhanced state surveillance and land seizure efforts. If these persist, legal exclusion will continue to shape the vulnerabilities of Indigenous peoples, second-generation immigrants, and other marginalized and minoritized peoples worldwide [4]. Thus, more analyses of LS adjudication and health, including through mechanisms involving education, are necessary in Thailand and elsewhere to extend this work to other contexts of bureaucratic violence and legal erasure.

Our findings also emphasize the importance of promoting human rights, education, and health as early in life as possible. Withholding any path to full citizenship rights at birth in contexts such as Thailand equates to withholding the full protection of one’s human rights, including health and education as rights. Recent efforts to address these problems include promoting universal birth registration campaigns as global health interventions. Yet, while birth registration is indeed associated with health advantages, our findings do not endorse interventions that prioritize registrations and documentation without clearly linking rights, and even citizenship, to registration. As evidenced in the ongoing experiences of highlanders in Thailand, even ostensibly inclusive and depoliticized registration campaigns can facilitate erasures and structural exclusions for generations to come, particularly when rights are not linked to, or even delinked from, registration status. Considering our study context and others, more careful design and interrogation of specific interventions and policy reform are still warranted. Ultimately, to intervene on legal exclusion as a structural determinant of health and inequities, considerations of how to expand and equalize health, education, and social service access in ways unobstructed by individual LS adjudication processes and related discrimination are also needed.

## Supplementary Information

**Additional file 1.**

## Data Availability

The data that support the findings of this study are available from the United Nations Educational, Scientific, and Cultural Organization (UNESCO) but restrictions apply to the availability of these data and so are not publicly available. Select data are however available from Dr. Amanda Flaim upon reasonable request and with permission of UNESCO.

## Abbreviations

HIV: Human immunodeficiency virus
AIDS: Acquired immunodeficiency syndrome
LS: Legal status
UNESCO: United Nations Educational, Scientific, and Cultural Organization
HPS: Highland Peoples Survey
CI: Confidence interval
