# Supplementary material for: Legal status as a life course determinant of health: parent status, adjudication stages, and HIV knowledge among highlanders in Thailand
Source: BMC Public Health. 2021 Oct 11;21:1839. doi: 10.1186/s12889-021-11811-8 (PMC8507192; doi:10.1186/s12889-021-11811-8)
Supplement: Supplementary file 1 — Additional file 1. [file 12889_2021_11811_MOESM1_ESM.pdf]

ONLINE SUPPLEMENTARY FILE

Methods supplement.
Figure A1: HIV questions and rates of correct responses by parent citizenship and ethnic minority status
Table A1: Missingness for analytical variables.
Table A2. Ordinal regression results.
Figure A2. Probability of HIV knowledge score ratios by parent citizenship.

Methods Supplement.

Ordinal Logistic Regressions

In order to examine the association between parents' citizenship status and HIV knowledge we modeled the respondents' relative odds of achieving above or below specific knowledge scores using a series of ordinal logistic regressions with random intercepts as follows:

$$\text{Log-odds (score} \geq \text{k)}_{ij} = \beta_{0kj} + \beta_1\text{(citizen parent)}_{ij} + \beta_2 x_{ij2} + \ldots$$

The dependent variable (score $\geq$ k) score was modeled as the log odds of answering *k* or more questions correctly, as compared to answering less than *k* questions correctly, for each value of *k*. An assumption of the model is that the odds of answering k or more questions correctly are proportional, or parallel, for all values of *k* (but not necessarily equal). We included random intercepts, β_{0kj}, in order to account for the influence of village characteristics on HIV knowledge. The fixed effect coefficient for a citizen parent (β_1) represents the log odds ratio for answering an additional question correctly, comparing individuals with and without a parent with citizenship.

Figure A1. Percentages of individual HIV knowledge questions answered correctly by subgroup: Thai with citizen parent (n=1,794), highlander with at least one citizen parent (n=5,761), and highlander without a citizen parent (n=2,318). Original questions asked whether each of the following were modes of HIV transmission: 1) sharing unclean needles, 2) having sex without a condom, 3) mother-to-child transmission (MCT) during pregnancy, 4) sharing food, 5) kissing, and 6) mosquito bites.

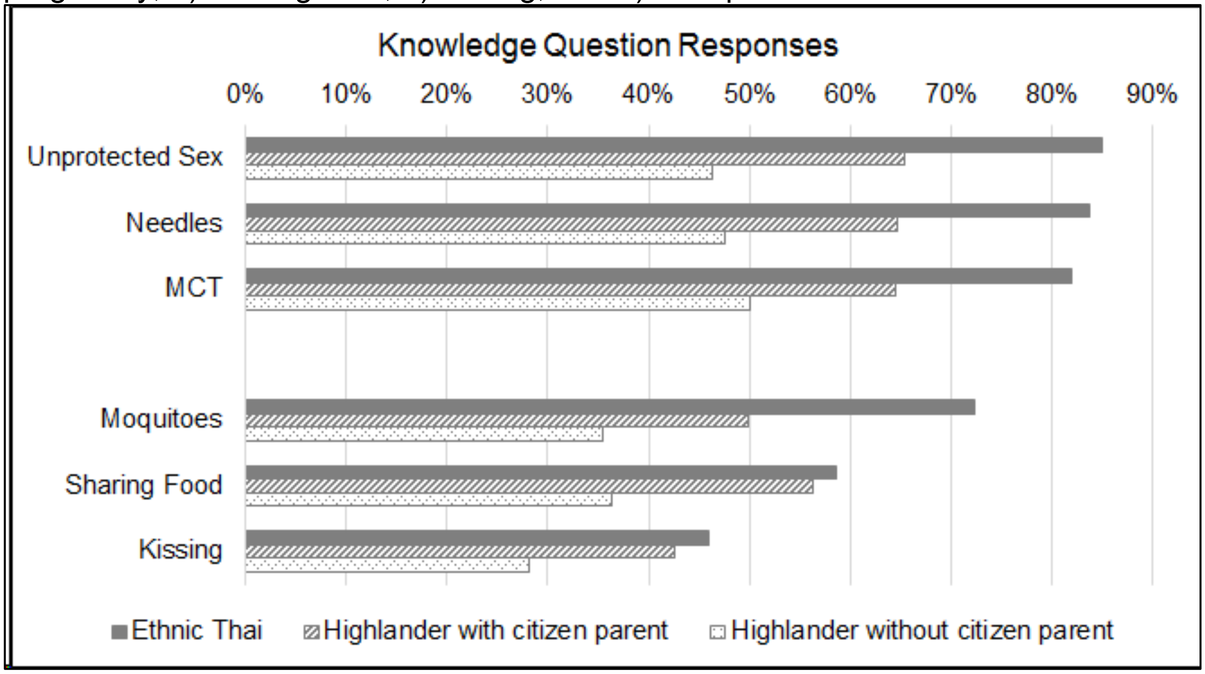

Table A1. Missingness for analytical variables.

	**Missing (n)**	**Percent**
HIV Knowledge Questions		
Unprotected Sex	362	3%
Needles	411	4%
Mother-to-child transmission	434	4%
Mosquitoes	445	4%
Sharing Food	452	4%
Kissing	517	5%
LS adjudication		
Parent with citizenship	440	4%
Birth registered	307	3%
Adult citizenship	89	1%
Education completed	50	0%
Age	0	0%
Male sex	49	0%
Ethnicity	0	0%
Wealth	37	0%

Table A2. ***Ordinal regression results.*** *Intercepts (log odds), coefficients (log odds ratios), and 95% confidence intervals (CI). Type III tests of fixed effects for each independent variable were statistically significant (p<0.01).*

	Point estimates	95% CI	
**HIV Score Intercepts**			
1	0.50	(0.05,	0.95)
2	0.33	(-0.12,	0.78)
3	-0.08	(-0.53,	0.37)
4	-0.81	(-1.26,	-0.36)
5	-1.47	(-1.92,	-1.02)
6	-2.58	(-2.58,	-2.13)
**Coefficients**			
**Parent citizenship**	0.62	(0.42,	0.82)
**Ethnic group**			
Karen	(Reference)		
Lahu	-0.67	(-1.18,	-0.16)
Akha	-0.18	(-0.71,	0.36)
Hmong	0.08	(-0.54,	0.70)
Lisu	-0.46	(-1.10,	0.18)
**Age group**			
Over 55	(Reference)		
46-55	0.22	(0.08,	0.36)
36-45	0.46	(0.33,	0.59)
26-35	0.89	(0.75,	1.02)
15-25	1.07	(0.91,	1.23)
**Sex**			
Male	0.36	(0.27,	0.45)
**Wealth**			
Wealth index	0.20	(0.16,	0.25)
**Parent citizenship * ethnicity interaction**			
Karen	(Reference)		
Lahu	-0.33	(-0.59,	-0.06)
Akha	-0.59	(-0.89,	-0.29)
Hmong	-0.48	(-0.90,	-0.06)
Lisu	-0.25	(-0.75,	0.26)

Figure A2. ***Probability of HIV knowledge score ratios by parent citizenship.*** *Predicted probabilities of each HIV score were estimated and used to calculate probability ratios based on parent citizenship (yes/no) among the three largest highlander ethnic minority groups: Karen, Lahu, and Akha. Estimates were recalculated across multiple model stages that added and conditioned on intermediate outcomes, including legal status adjudication stages and education. Stages started with parent citizenship (PC) alone and covariates (age, age^2, age^3, and sex). Subsequent stages then added and conditioned on the following intermediate variables and respective model terms, represented in separate lines: (1) birth registration (BR) and BR*PC interaction; (2) secondary education complete (ED) and ED*PC interaction; and (3) adult citizenship documentation (C) and C*PC interaction.*

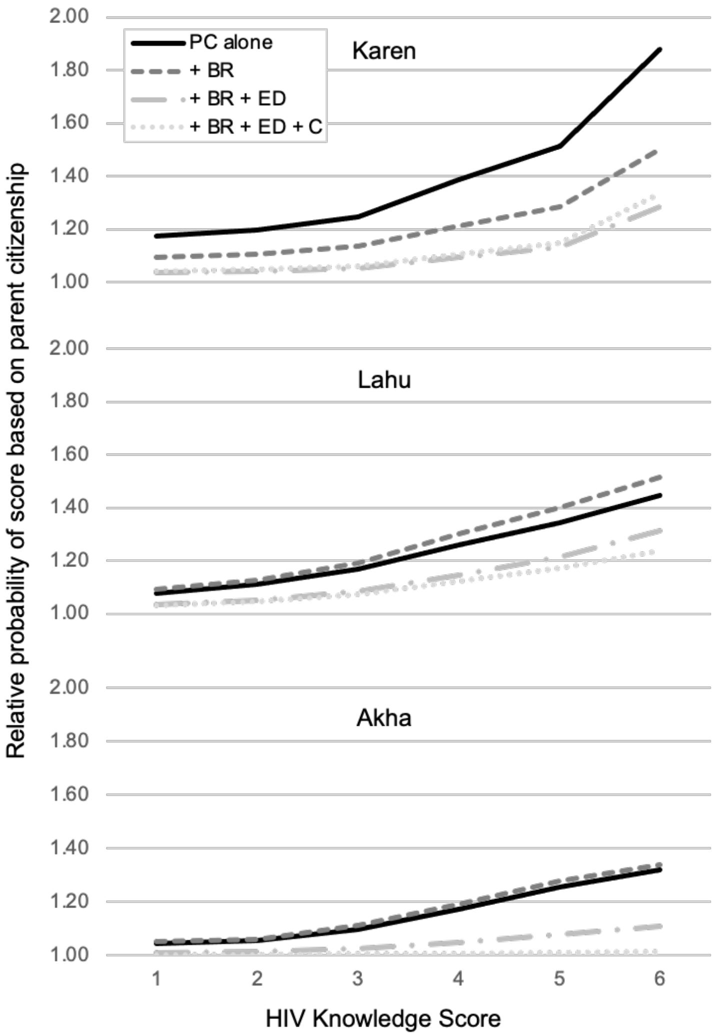

Figure A2 presents model predictions from a staged models paralleling our original model (results summarized in Table 2) but with slightly fewer assumptions. In the models used to predict the above estimates we allow for the associations between HIV knowledge and intermediate legal status and educational outcomes to vary by parent citizenship. This simply allows us to plot patterns closer to how they appear in the unadjusted data, while still adjusting for other confounders (specifically, age and sex).

Figure A2 depicts how the association between parent citizenship and HIV knowledge is attenuated by intermediate LS adjudication and education across HIV score values. This visualization highlights how HIV score differences are greatest at higher (*i.e.*, more difficult) score values. It also highlights differences in the differences scores by parent citizenship, and relative attenuation by intermediate outcomes, across ethnic groups. Among the Karen, parent citizenship accounts for the largest difference in high HIV scores (over 80% higher probability of a perfect score), which is partially accounted for by birth registration and adult citizenship. After controlling for these factors, parent citizenship is still associated with over a 20% greater probability of answering all six questions correctly. Among the Lahu, parent citizenship is associated with a 50% higher probability of a perfect score, which is most attenuated by education but only partially. After accounting for all intermediate variables, parent citizenship is still associated with over a 20% greater probability of a perfect score. Finally, among the Akha, a perfect score is at least 30% more likely among respondents with a citizen parent. This is attenuated by two-thirds after accounting for education and completely disappears after accounting for adult citizenship. This suggests that parent citizenship advantage is more fully mediated through education and intermediate LS among Akha than among other groups.
